# Single Nucleotide Polymorphisms of *FAM13A* Gene in Chronic Obstructive Pulmonary Disease—A Case Control Study in Vietnam

**DOI:** 10.3390/arm91030021

**Published:** 2023-06-15

**Authors:** Khanh Hoang Pham, Nhung Thi Cam Tran, Hung Do Tran, Toan Hoang Ngo, Van De Tran, Hung Huynh Vinh Ly, Nga Thi Ngoc Pham, Thang Nguyen, Binh Huy Nguyen, Kien Trung Nguyen

**Affiliations:** 1 Faculty of Medicine, Can Tho University of Medicine and Pharmacy, Can Tho City 900000, Vietnam; phkhanh@ctump.edu.vn (K.H.P.); tdhung@ctump.edu.vn (H.D.T.); nhtoan@ctump.edu.vn (T.H.N.); tvde@ctump.edu.vn (V.D.T.); 1853010822@student.ctump.edu.vn (H.H.V.L.); ptnnga@ctump.edu.vn (N.T.N.P.); nthang@ctump.edu.vn (T.N.); 2 Department of Anesthesiology and Resuscitation, Hoan My Cuu Long Hospital, Can Tho City 900000, Vietnam; ttcnhungoxy@gmail.com; 3 Physiology Department, Hanoi Medical University, Ha Noi 100000, Vietnam; nguyenhuybinh@hmu.edu.vn

**Keywords:** chronic obstructive pulmonary disease, *FAM13A*

## Abstract

**Highlights:**

**What are the main findings?**

- Study and investigation of the association of single nucleotide polymorphisms (SNPs) in the *FAM13A* gene with COPD.
- Determination of the allele frequency and genotype phenotypes of rs2869967 and rs17014601 in the *FAM13A* gene in individuals with COPD, and investigation of the phenotypic association with COPD risk.

**What is the implication of the main finding?**

- Further studies should be conducted on nucleotide polymorphisms in the *FAM13A* gene to understand the relationship between SNP polymorphisms and respiratory function parameters, and to continue the progress towards constructing a predictive model for the severity of COPD with *FAM13A* gene SNP polymorphisms.
- Future directions include constructing a map of human gene polymorphisms in Vietnam, focusing on the study of genes impacting chronic obstructive pulmonary disease (COPD).

**Abstract:**

Background: In 2018, GOLD addressed the issues of genotypes associated with risk factors for COPD. The genome-wide association study (GWAS) demonstrated an association between COPD and several genetic variants of single nucleotide polymorphisms (SNPs) of the *FAM13A* gene with the risk of COPD. Objective: To study the single nucleotide polymorphisms rs2869967 and rs17014601 of the *FAM13A* gene in chronic obstructive pulmonary disease. Subjects and research methods: 80 subjects diagnosed with COPD and 80 subjects determined not to have COPD according to GOLD 2020 criteria; the subjects were clinically examined, interviewed, and identified as possessing single nucleotide polymorphisms using the sanger sequencing method on whole blood samples. Results: The male/female ratio of the patient group and the control group was 79/1 and 39/1, respectively. The percentages of C and T alleles of rs2869967 in COPD patients were 50.6% and 49.4%, respectively. The percentages of C and T alleles of rs17014601 in COPD patients were 31.9% and 68.1%, respectively. At rs17014601, the ratio values of alleles T and C in the disease group and the control group were markedly different, making them statistically reliable (*p* = 0.031). The rate of CT genotype in the group of patients was considerably higher than that of the control group. The TT homozygous genotype had a lower risk of COPD compared with the other genotypes in the dominant model (ORTT/(CC + CT) = 0.441; CI95% = 0.233–0.833); this difference was statistically significant (*p* = 0.012). Conclusions: With rs17014601, it is characteristic that the frequency of the T allele appears more than the C allele, and the CT heterozygous phenotype accounts for the highest proportion in rs17014601 and rs2869967 recorded in COPD patients. There is an association between the genetic variant of the SNP *FAM13A*-rs17014601 and the risk of COPD.

## 1. Introduction

Chronic obstructive pulmonary disease (COPD) is a common respiratory disease characterized by chronic obstruction of the airways [1,2]. According to WHO, this disease will be the third leading cause of death in the world by 2030. According to the Asia-Pacific Respiratory Society, the morbidity of COPD in Vietnam is 6.7%, which is the highest among the 12 countries in this region. Genotypic issues with COPD risk have been previously addressed by GOLD in 2018. Currently, research on genes in COPD is receiving increasing amounts attention and research. In the 21st century, many studies have shown that genetic traits influence the development of COPD [3,4,5,6,7,8,9,10]. In particular, studies have mentioned the role of *FAM13A* in the regulation of CPT1A (Carnitine palmitoytransferase-1A) and FAO (Fatty acid oxidation). It was found from previous studies that *FAM13A* combined with SIRT1 (Sirtuin-1) intermediate protein to control the expression of CPT1A, which is an important enzyme that regulates fatty acid oxidation (FAO) in mitochondria. Increasing CPT1A promotes fatty acid oxidation (FAO), which, in turn, leads to increased production of ROS (Reactive oxygen species), which are reactive oxygen species derived from oxygen including free radicals and some other specific molecules, resulting in the death of lung epithelial cells, thereby forming the irreversible alveolar damage of COPD [7,11].

Especially recently, there have been many studies on the *FAM13A* gene in COPD and lung function [12,13]; typically, the results of a genome-wide association study (GWAS) have demonstrated a statistically significant association between COPD and some polymorphisms. Single nucleotides (SNPs) of the *FAM13A* gene [8], such as rs7671167, rs10007590, rs2869966, rs2869967 and rs17014601 are associated with an increased risk of COPD. Among these SNPs, rs2869967, and rs17014601 have been consistently associated with the risk of COPD in most studies [4,6,10,14]. We conducted a study on 80 subjects in the COPD group and 80 in the control group (without COPD) who visited the Hospital of Can Tho University of Medicine and Pharmacy. The primary objective was to study and investigate the association of single nucleotide polymorphisms (SNPs) of the *FAM13A* gene in COPD. Then, the secondary objective was to determine the allele frequency and genotype phenotypes of rs2869967, and rs17014601 in the *FAM13A* gene in COPD, and investigate the phenotypic association with COPD risk.

## 2. Materials and Methods

### 2.1. Study Population

This study was conducted according to the case–control study method with 160 outpatients, who were selected via purposive sampling, selecting those who met the sampling criteria set forth by the research objective. The research data collected have 80 outpatients in the disease group, and 80 outpatients in the control group, who came to the hospital of Can Tho University of Medicine and Pharmacy with stable clinical conditions and agreed to participate in the study. The study selected outpatients in the disease group who were all COPD patients diagnosed according to GOLD 2020 criteria with stable clinical conditions. The control group was outpatients who came to the clinic and were determined not to have COPD according to GOLD 2020 criteria, without chronic respiratory diseases and agreed to participate in the study. All patients selected in this study in both groups were those who consented to participate in the study. This study also excluded patients who did not conform to the criteria, including patients with severe respiratory conditions such as pneumothorax, hemoptysis, pulmonary embolism, etc.; patients with advanced malignancy; patients with multiple comorbidities; and patients who did not consent to participate in the study.

### 2.2. Research Content

**Figure 1:**
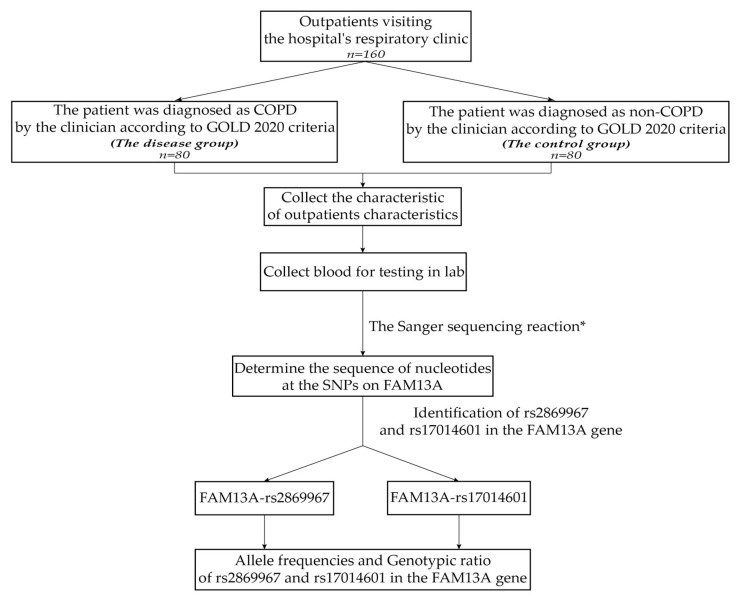
Research performance flowchart. * The Sanger sequencing reaction was used to investigate mutations, and capillary electrophoresis was conducted on the ABI 3500 Genetic Analyzer system. The PCR product can be utilized for the next PCR-RFLP technique to determine the sequence of nucleotides at the SNPs on *FAM13A*.

This study recorded the general information characteristics of the study subjects including age, sex, height, weight, BMI, education level, pulse, systolic and diastolic blood pressure. The single nucleotide polymorphisms rs2869967 and rs17014601 of the *FAM13A* gene were determined via Sanger sequencing on whole blood specimens at the Center for Molecular Biomedical Medicine of the University of Medicine and Pharmacy in Ho Chi Minh City, Vietnam (Figure 1 describes the process of data collection and analysis in this study).

Primer pairs designed on the *FAM13A* gene include *FAM-967F*/*FAM-966R* and *FAM-601F*/*FAM-601R* with sizes of 408 bp and 213 bp, specifically presented in Table 1. This study also determined allele frequencies and genotypes of *FAM13A,* and analyzed the association with the risk of COPD.

Through a set of patient interview questions, clinical examination of each patient, and collection of whole blood samples to send for *FAM13A* gene testing, we have a baseline data source for analysis.

### 2.3. Data Analysis

After collecting the necessary information, the data were analyzed using SPSS software version 18.0. The Kolmogorov–Smirnov test and the normal Q-Q plot are used to assess the normality of the data. We used the chi-square test (χ^2^) and Fisher’s exact test to compare the proportions between two or more groups; *t*-test (for normally distributed data) and the Mann–Whitney test (for non-normally distributed data) were used to test the significance of any association or difference between two independent samples at the level of statistical significance of *p*-value ≤ 0.05. The Hardy–Weinberg equilibrium (HWE) test was applied to confirm the independent segregation of the alleles.

## 3. Results

### 3.1. Clinical Characteristics of the Study Population

This study involved 160 subjects, 80 of which were in the disease group, with the remaining 80 subjects being in the control group. The general characteristics of the study subjects are described in Table 2 and Table 3. The male/female ratios of the patient group and the control group were 79/1 and 39/1, respectively. The group of patients with primary school level accounted for the highest rate of 46.3%. The control group with a level equivalent to secondary level constituted the highest rate of 42.5%. The average age, height, and BMI of the study group were 66.34 ± 7.90; 162.31 ± 5.81; 22.52 ± 3.57, respectively. Similarly, the mean values of pulse and blood pressure (SYS and DIA) were 86.29 ± 10.98, 137.28 ± 13.06 and 88.29 ± 8.29, respectively. Overall, the mean values of the disease group are higher than the control group.

### 3.2. Identification of rs2869967 and rs17014601 in the FAM13A Gene

**Figure 2:**
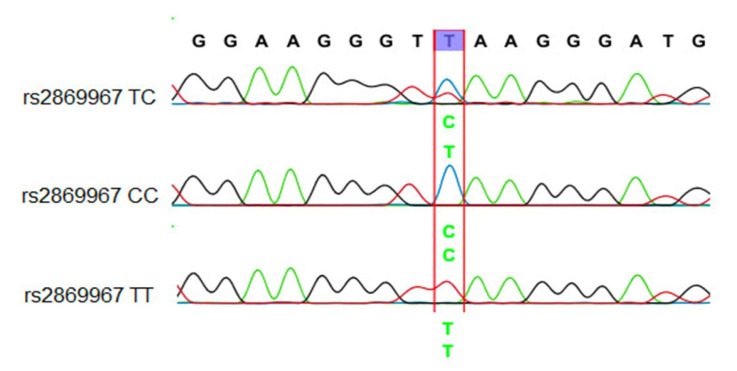
A part of the nucleotide sequence of the *FAM13A* rs2869967 SNP.

**Figure 3:**
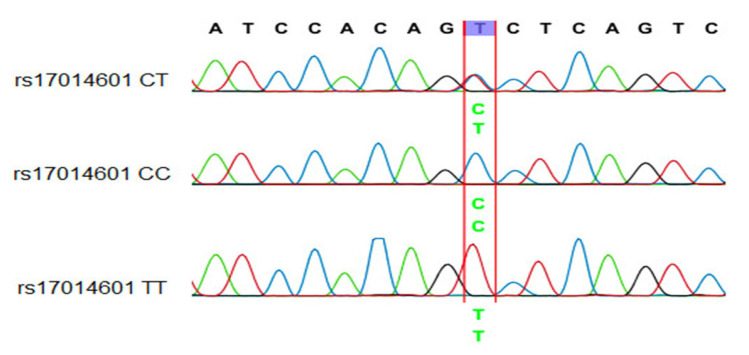
A part of the nucleotide sequence of the *FAM13A* rs17014601 SNP.

The Sanger sequencing reaction was used to investigate mutations, and capillary electrophoresis was conducted on the ABI 3500 Genetic Analyzer system. The PCR product can be utilized for the next PCR-RFLP technique to determine the sequence of nucleotides at the SNPs on *FAM13A*. Figure 2 and Figure 3 are the results of sequencing PCR products containing SNP *FAM13A*-rs2869967 and SNP *FAM13A*-rs17014601.

The results of sequencing PCR products containing SNP *FAM13A*-rs2869967 found that the sequencing signals witness clear nucleotide peaks. The PCR sample of genotype CC has a single peak of nucleotide C, genotype CT has two peaks of nucleotide C and nucleotide T, and genotype TT has a single peak of nucleotide T. As a consequence, the sequencing results and the RFLP genotyping results obtained are comparable.

In PCR products containing SNP *FAM13A*-rs17014601, the recorded nucleotide peaks are clearly visible in the sequencing data. The PCR sample of genotype CC has a single peak of nucleotide C, genotype CT has two peaks of nucleotide C and nucleotide T, and genotype TT has a single peak of nucleotide T. Thus, the collected sequencing results and the RFLP genotyping results are similar.

### 3.3. Allele Frequencies of rs2869967 and rs17014601 in the FAM13A Gene

Frequency analysis of C and T alleles of rs2869967 and rs17014601 in the *FAM13A* gene recorded that the disease and control groups had 50.6% and 47.5% of the C allele of rs2869967, respectively. The proportion of T allele of rs2869967 in the disease and control groups was 49.4% and 52.5%, respectively. The percentage of C allele of rs17014601 in the disease group was 31.9% and that in the control groups was 21.3%, respectively. The percentages of T allele of rs17014601 in the disease and control groups were 68.1% and 78.8%, respectively. In SNP *FAM13A*-rs17014601, the ratio values of alleles T and C in the disease group and the control group were statistically significant (Table 4).

### 3.4. Genotypic Ratio of rs2869967 and rs17014601 in the FAM13A Gene

In rs2869967, the genotype frequencies in the control and disease group were in Hardy–Weinberg equilibrium (X^2^ = 1.869, *p* = 0.171 and X^2^ = 0.452, *p* = 0.501). Similarly, for rs17014601, the values were (X^2^ = 2.544, *p* = 0.110 in the disease group and X^2^ = 0.337, *p* = 0.561 in the control group), indicating that rs17014601 also conforms to the Hardy–Weinberg equilibrium.

Table 5 shows the genotypic ratio of *FAM13A*-rs2869967. The CC homozygous genotype had a higher risk of COPD than the remaining genotypes in the recessive inheritance model (ORCC/(TT + CT) = 1.350; CI 95% = 0.630–2.891). The TT homozygous genotype had a lower risk of chronic obstructive pulmonary disease than the other genotypes in the dominant inheritance model (ORTT/(CC + CT) = 0.932; CI 95% = 0.446–1.944).

For *FAM13A*-rs17014601, the rate of CT genotype in the disease group recorded was higher than that in the control group, which was statistically significant (Table 6). The homozygous TT genotype had a lower risk of COPD than the other genotypes in the dominant model (ORTT/(CC + CT) = 0.441; CI 95% = 0.233–0.833); this difference had statistical significance (*p* = 0.012).

## 4. Discussion

This study recorded a majority of male patients (98.8%), showing that COPD predominates in men; this finding is similar to that of Nguyen CT’s study, the rate of which for males is 88.4% [15]. The average age of the COPD patients was 66.71 ± 7.93, which is comparable to Kraen M’s research with an average age of 66.0 ± 6.2 [16] and similar to another study in Can Tho [17]. As a result, our anthropometric epidemiological study’s results are consistent with the characteristics of COPD, which usually occurs in men with an average age of 60 years or more. The mean height and overall BMI of the study group were 162.31 ± 5.81 cm and 22.52 ± 3.57 kg/m^2^, respectively. This result is similar to the average height reported by the Ministry of Health of Vietnam in 2020, which is 168.1 cm in men and 156.2 cm in women [15]. The overall BMI value of our study falls within the ideal BMI group based on the classification scale of IDI & WPRO for Asian people.

### 4.1. Allele Frequencies of rs2869967 and rs17014601 in the FAM13A Gene

In rs2869967, our study results had a higher rate of the C allele in the disease group than in the control group and vice versa in that of T allele (Table 3). In addition, we also observed results in the disease group with the frequency of allele C being higher than that of T; these results are entirely consistent with those of Gou’s study and those of the study of Wang [5,8]. Specifically, Gou recorded that the C allele in the group disease was higher than that of the control group (0.555 and 0.5), and the T allele was lower in the disease group (0.455 and 0.5). In addition, Wang recorded that the ratio of the C allele was 0.515 and 0.493 in the disease group and the control group, respectively, resulting in the T allele being lower in the disease group (0.485 and 0.507) [8]. However, our study’s author Wang did not find a statistically significant difference, while Gou’s study found it to be statistically significant (*p* = 0.042) [5].

Regarding rs17014601 in our study, we found a statistically significant difference in the frequency of C and T alleles in the disease group and the control group (*p* = 0.031), showing an association between polymorphic variant single nucleotide rs17014601 of the *FAM13A* gene and COPD risk in Vietnam. This result is similar to that of Yanan Zhang’s study, which showed that there was a difference in the ratio of T > C allele between the disease group and the control group, which proved that rs17014601 is closely correlated with an increased risk of chronic obstructive pulmonary disease [9].

### 4.2. Genotypic Ratio of rs2869967 and rs17014601 in the FAM13A Gene and Its Association with COPD Risk

This study analyzed the association between single nucleotide polymorphism (SNP) of the *FAM13A* gene and COPD in two types of SNPs, *FAM13A*-rs2869967, and *FAM13A*-rs17014601. Specifically, in rs2869967, the CC homozygous genotype had a higher risk of COPD compared with the remaining genotypes in the recessive inheritance model; additionally, the homozygous TT genotype had a lower risk of chronic obstructive pulmonary disease than the other genotypes in the dominant inheritance model. This result is similar to those of Wang’s study, which recorded the CC genotype with the highest risk of COPD and the TT genotype with the lowest risk. However, our study did not find a statistically significant association of rs2869967 with COPD risk [8].

Meanwhile, in the gene *FAM13A*-rs17014601, the heterozygous CT genotype accounted for the highest proportion in the disease group (46.3%); this result is similar to those of Zhang and colleagues’ study (48.4%). Our study’s and Zang’s findings, in particular, share the fact that these values all demonstrate a statistically significant difference when compared to the control group [9]. In addition, in our study, it was found that the homozygous TT genotype had a lower risk of COPD compared with other genotypes in the dominant inheritance model (OR = 0.441, 95% CI [0.233–0.833], *p* = 0.012).

Analysis of multivariate logistic regression between COPD and rs2869967, rs17014601 of the *FAM13A* gene, age, gender, and BMI demonstrated that individuals with the CT genotype have a higher risk of developing COPD compared to individuals with other rs17014601 genotypes (Table 7).

### 4.3. Implication and Further Work

This study identified a single nucleotide polymorphism of the *FAM13A* gene. However, the initial study only identified two SNPs with the risk of COPD but did not study the relationship between SNPs and impaired lung function. Other SNPs in the *FAM13A* gene were not considered in this study. Therefore, the research team will continue to learn more about the remaining SNPs and further explore the role of SNPs phenotypes in the *FAM13A* gene and the status of pulmonary ventilation disorders.

## 5. Conclusions

The proportion of C and T alleles of rs2869967 and rs17014601 in COPD patients was 50.6%, 49.4%, and 31.9%, 68.1%, respectively. The CT heterozygous phenotype accounted for the highest proportion in rs17014601 and rs2869967. This study showed an association between the genetic variant of the SNP *FAM13A*-rs17014601 and the risk of COPD.

## Figures and Tables

**Table 1:** Primer pairs designed on the *FAM13A* gene.

Name of Primer	The Sequence of Nucleotides (3′–5′)	Length (bp)	PCR Size (bp)
FAM-967F	CCTACACTATATGAGTTGTG	20	408
FAM-966R	ATAGATATTCTCAGGCCTTG	20	
FAM-601F	GACCAAACCAAAAACCTAAG	20	213
FAM-601R	ACTCAGGCATTTTCCACATG	20	

Note: PCR = polymerase chain reaction; bp = base pair.

**Table 2:** Genders and education level of the patients.

Groups		Disease Group (*n* = 80)	Control Group (*n* = 80)	*p*
Characteristics		*n* (%)	*n* (%)	
Gender	Male	79 (98.8)	78 (97.5)	1.000
	Female	1 (1.2)	2 (2.5)	
Education	Primary	37 (46.3)	19 (23.8)	0.009
	Secondary	21 (26.3)	34 (42.5)	
	High school and tertiary	22 (27.5)	27 (33.8)	

**Table 3:** Age, anthropometric, pulse and blood pressure characteristics of the patients.

Characteristics	Disease Group (*n* = 80)	Control Group (*n* = 80)	Total (*n* = 160)	*p* *
Age (years)	66.71 ± 7.93	65.96 ± 7.91	66.34 ± 7.90	0.525
Height (cm)	162.70 ± 6.24	161.91 ± 5.34	162.31 ± 5.81	0.167
Weight (kg)	58.91 ± 9.84	59.69 ± 9.52	59.30 ± 9.66	0.613 **
BMI (kg/m^2^)	22.27 ± 3.65	22.77 ± 3.49	22.52 ± 3.57	0.379 **
Pulse (times/min)	86.86 ± 11.12	85.73 ± 10.87	86.29 ± 10.98	0.475
SYS (mmHg)	138.23 ± 14.42	136.34 ± 11.56	137.28 ± 13.06	0.436
DIA (mmHg)	88.40 ± 9.41	88.18 ± 7.04	88.29 ± 8.29	0.843

Note: BMI = Body Mass Index; SYS = Systole; DIA = Diastole; * Mann–Whitney test; ** *t*-test.

**Table 4:** C and T allele frequencies of rs2869967 and rs17014601 in the *FAM13A*.

Allele		Disease Group		Control Group		*p*
		*n*	%	*n*	%	
*FAM13A*-rs2869967	C	81	50.6%	76	47.5%	0.576
	T	79	49.4%	84	52.5%	
*FAM13A*-rs17014601	C	51	31.9%	34	21.3%	0.031
	T	109	68.1%	126	78.8%	

**Table 5:** Genotypic ratio of rs2869967 in *FAM13A* gene.

*FAM13A*-rs2869967		Disease Group		Control Group		*p*	OR (CI 95%)
		*n*	%	*n*	%		
Genotypes	TT	18	22.5%	19	23.8%	1	1
	CT	43	53.8%	46	57.5%	0.973	0.987 (0.458–2.125)
	CC	19	23.8%	15	18.8%	0.543	1.337 (0.525–3.405)
Recessive Inheritance	CC	19	23.8%	15	18.8%	0.440	1.350 (0.630–2.891)
	TT + CT	61	76.2%	65	81.3%		1
Dominant Inheritance	TT	18	22.5%	19	23.8%	0.851	0.932 (0.447–1.944)
	CT + CC	62	77.5%	61	76.3%		1

Note: OR = odds ratio; CI = confidence interval.

**Table 6:** Genotypic ratio of rs17014601 in *FAM13A* gene.

*FAM13A*-rs17014601		Disease Group		Control Group		*p*	OR (CI 95%)
		*n*	%	*n*	%		
Genotypes	TT	36	45.0%	52	65.0%	1	1
	CT	37	46.3%	22	27.5%	0.010	2.429 (1.234–4.783)
	CC	7	8.8%	6	7.5%	0.382	1.685 (0.523–5.431)
Recessive Inheritance	CC	7	8.8%	6	7.5%	0.773	1.183 (0.379–3.688)
	TT + CT	73	91.3%	74	92.5%		1
Dominant Inheritance	TT	36	45.0%	52	65.0%	0.012	0.441 (0.233–0.833)
	CT + CC	44	55.0%	28	35.0%		1

Note: OR = odds ratio; CI = confidence interval.

**Table 7:** A multivariate logistic between rs2869967 and rs17014601 of the *FAM13A* in COPD with age, gender, and BMI.

Variables	B	S.E.	Wald	df	Sig.	Exp (B)	95% CI
Gender	−0.783	1.282	0.373	1	0.541	0.457	0.037–5.64
Age	0.011	0.021	0.29	1	0.590	1.011	0.971–1.054
BMI	−0.046	0.047	0.929	1	0.335	0.955	0.871–1.048
rs2869967							
TT			0.437	2		1	
CT	0.046	0.405	0.013	1	0.909	1.047	0.474–2.316
CC	0.297	0.496	0.358	1	0.550	1.345	0.509–3.557
rs17014601							
TT			7.02	2		1	
CT	0.911	0.349	6.796	1	0.009	2.486	1.254–4.931
CC	0.666	0.616	1.167	1	0.280	1.946	0.581–6.513
Constant	0.603	2.288	0.07	1	0.792	1.828	

Note: B = coefficient estimate; S.E. = standard error; Wald = Wald statistic; df = degree of freedom; Sig = significance level; Exp (B) = exponential; CI = confidence interval.

## Data Availability

The datasets generated and/or analyzed during the current study are available from the corresponding author on reasonable request.
